# Supplementary material for: End Sequence Analysis Toolkit (ESAT) expands the extractable information from single-cell RNA-seq data
Source: Genome Res. 2016 Oct;26(10):1397–410. doi: 10.1101/gr.207902.116 (PMC5052061; doi:10.1101/gr.207902.116)
Supplement: Supplemental Material [file supp_gr.207902.116_Supp_Materials.tar.gz › ESAT-esat_v0.1/doc/html/classumms_1_1core_1_1alignment_1_1_smatch_like.html]

ESAT: umms.core.alignment.SmatchLike Class Reference


ESAT


- Main Page
- Related Pages
- Packages
- Classes
- Files

- Class List
- Class Index
- Class Hierarchy
- Class Members

All Classes Namespaces Files Functions Variables Enumerator Pages

- umms
- core
- alignment
- SmatchLike

Public Member Functions |
Static Public Member Functions |
List of all members

umms.core.alignment.SmatchLike Class Reference

Collaboration diagram for umms.core.alignment.SmatchLike:

[legend]

|  |  |
| --- | --- |
| Public Member Functions | |
|  | SmatchLike (String kmer, List< Sequence > geneSequences, int seedNum) |
|  | |
|  | SmatchLike (String kmer, String primer, int seedNum) |
|  | |
|  | SmatchLike (String kmer2, Collection< Pair< String >> tailPrimers, Pair< String > subprimer1) |
|  | |
| Collection< String > | getForwardTargets (int numMismatches) throws SearchException |
|  | |
| Map< String, List  < SequenceRegion > > | getForwardTargetRegions (int numMismatches) throws SearchException |
|  | |
| Collection< String > | getReverseTargets (int numMismatches) throws SearchException |
|  | |
| Collection< String > | getAllPossibleTargets (int numMismatches) throws SearchException |
|  | |
| int | numPossibleTargets () |
|  | |

|  |  |
| --- | --- |
| Static Public Member Functions | |
| static void | main (String[] args) throws IOException, SearchException |
|  | |

## Constructor & Destructor Documentation

|  |  |  |  |
| --- | --- | --- | --- |
| umms.core.alignment.SmatchLike.SmatchLike | ( | String | *kmer*, |
|  |  | List< Sequence > | *geneSequences*, |
|  |  | int | *seedNum* |
|  | ) |  |  |

Here is the caller graph for this function:

|  |  |  |  |
| --- | --- | --- | --- |
| umms.core.alignment.SmatchLike.SmatchLike | ( | String | *kmer*, |
|  |  | String | *primer*, |
|  |  | int | *seedNum* |
|  | ) |  |  |

|  |  |  |  |
| --- | --- | --- | --- |
| umms.core.alignment.SmatchLike.SmatchLike | ( | String | *kmer2*, |
|  |  | Collection< Pair< String >> | *tailPrimers*, |
|  |  | Pair< String > | *subprimer1* |
|  | ) |  |  |

## Member Function Documentation

|  |  |  |  |  |  |
| --- | --- | --- | --- | --- | --- |
| Collection<String> umms.core.alignment.SmatchLike.getAllPossibleTargets | ( | int | *numMismatches* | ) | throws SearchException |

Here is the call graph for this function:

|  |  |  |  |  |  |
| --- | --- | --- | --- | --- | --- |
| Map<String, List<SequenceRegion> > umms.core.alignment.SmatchLike.getForwardTargetRegions | ( | int | *numMismatches* | ) | throws SearchException |

|  |  |  |  |  |  |
| --- | --- | --- | --- | --- | --- |
| Collection<String> umms.core.alignment.SmatchLike.getForwardTargets | ( | int | *numMismatches* | ) | throws SearchException |

|  |  |  |  |  |  |
| --- | --- | --- | --- | --- | --- |
| Collection<String> umms.core.alignment.SmatchLike.getReverseTargets | ( | int | *numMismatches* | ) | throws SearchException |

Here is the call graph for this function:

|  |  |  |  |  |  |
| --- | --- | --- | --- | --- | --- |
| static void umms.core.alignment.SmatchLike.main | ( | String[] | *args* | ) | throws IOException, SearchException |

static

Here is the call graph for this function:

|  |  |  |  |  |
| --- | --- | --- | --- | --- |
| int umms.core.alignment.SmatchLike.numPossibleTargets | ( |  | ) |  |

---

The documentation for this class was generated from the following file:

- java/umms/core/alignment/SmatchLike.java


---

Generated on Fri Aug 15 2014 15:11:07 for ESAT by  

 1.8.7
